# Chimeric antigen receptor T-cell therapy in relapsed or refractory mantle cell lymphoma: a systematic review and meta-analysis

**DOI:** 10.3389/fimmu.2024.1435127

**Published:** 2024-09-06

**Authors:** Haixiang Wan, Songqin Weng, Sumei Sheng, Zilin Kuang, Qingming Wang, Linhui Hu

**Affiliations:** Jiangxi Provincial Key Laboratory of Hematological Diseases, Department of Hematology, The 2nd Affiliated Hospital, Jiangxi Medical College, Nanchang University, Nanchang, Jiangxi, China

**Keywords:** CAR-T, relapsed or refractory, mantle cell lymphoma, meta-analysis, therapy

## Abstract

**Background:**

Chimeric antigen receptor (CAR) T-cell therapy (CAR-T therapy) has demonstrated significant efficacy in the ZUMA-2 study. After regulatory approvals, several clinical trials and real-world studies on CAR-T therapy for relapsed or refractory mantle cell lymphoma (R/R MCL) were conducted. However, data on clinical safety and efficacy are inconsistent. In this study, we aimed to conduct a systematic analysis of the effectiveness and safety of CAR-T therapy across a wider and more representative cohort of patients with R/R MCL.

**Methods:**

We performed a systematic review and meta-analysis of studies on patients with R/R MCL who received CAR-T cell therapy. Data were extracted and consolidated, with primary focus on the evaluation of safety and efficacy outcome measures. This study has not been registered with PROSPERO.

**Results:**

This meta-analysis identified and included 16 studies with 984 patients. The pooled estimate for overall response rate (ORR) was 89%; complete remission (CR) rate was 74%. The 6-month and 12-month progression-free survival (PFS) rates were 69% and 53%, respectively, while the overall survival (OS) rates were 80% and 69%, respectively. Cytokine release syndrome (CRS) of grade 3 or higher was observed in 8% of patients, whereas neurotoxicity of grade 3 or higher was observed in 22% of patients. The risk of bias was assessed as low in 9 studies and moderate in 7 studies.

**Conclusion:**

CAR-T therapy exhibited promising efficacy and manageable adverse reactions in patients with R/R MCL.

## Introduction

Mantle cell lymphoma (MCL) is a distinct, rare subtype of B-cell non-Hodgkin lymphoma (representing approximately 2.5%–6% of total cases) (1). The clinical course of the disease is heterogeneous, ranging from indolent forms that may not require treatment for years to highly aggressive variants that carry a grave prognosis despite intensive therapeutic regimens (2). Particularly for patients with MCL with high-risk disease profiles, including those with blastoid variants, elevated Ki-67 proliferation indices, *TP53* gene mutations or increased protein expression, and disease progression within 24 months of initial diagnosis, the prognosis is generally poor (3).

Although Bruton’s tyrosine kinase inhibitors (BTKis) have significantly improved clinical outcomes for patients with MCL, the average progression-free survival (PFS) after BTK treatment remains unsatisfactory at 16.4 months. This suggested that most patients exhibit early disease progression through second line treatment. For individuals who experience disease after BTKi treatment failure, the prognosis is particularly severe, with a median overall survival (OS) of only 2.9 months. Treatment of patients with R/R MCLwho are resistant to BTKi therapy remains challenging (4–6).

Recent years have witnessed a significant transformation in the therapeutic landscape for R/R MCL (7, 8). A shift from traditional chemotherapy and immunotherapy to advanced targeted and cellular therapies, particularly CAR-T therapy, marks a new era in treatment modalities (9, 10). Groundbreaking results from the ZUMA-2 and TRANSCEND NHL 001 trials highlight the efficacy of CAR-T therapy in relapsed and drug-resistant patients with MCL (11–13). This therapy exhibits notable response rates in subsets of patients characterized by advanced age, blastoid phenotypes, elevated Ki-67 proliferation indices, high MIPI scores, and *TP53* mutations, as well as those with central nervous system involvement (12). Though CAR-T therapy demonstrates strong antitumor efficacy in relapsed or refractory B-cell hematologic malignancies, it is imperative to acknowledge its adverse effects including neurotoxicity, cytokine release syndrome (CRS), on-target/off-tumor recognition, and insertional oncogenesis (14, 15).

Approval by FDA in July 2020 and subsequent endorsement by European medical authorities in January 2021 for the use of CD19 CAR-T cells in patients with R/R MCL signifies a crucial step forward. As CAR-T therapy gains momentum in clinical application and long-term follow-up data are accumulated, it is imperative to study safety and efficacy evidence. In this study, we aimed to meticulously review the existing trials of CAR-T therapy for MCL, to evaluate the clinical outcomes and toxicity profiles rigorously, and to determine the factors that influence divergent treatment responses.

## Methods

### Search strategy and selection criteria

We conducted literature searches across multiple databases including PubMed, Embase, Cochrane Systematic Reviews, and ClinicalTrials.gov. Data were collected up to July 4, 2024. The search strategy employed the terms “CAR-T” and “mantle cell lymphoma,” with the detailed methodology outlined in the **Supplementary File 1**. The inclusion criteria were studies with patients with MCL undergoing CAR-T therapy, without any restrictions on date or study design. Exclusion criteria included studies without complete data, basic research, case series with fewer than 10 patients, and studies on dual targets, which were discussed separately.

In this meta-analysis, key data including demographic information, treatment history, and clinical outcomes of CAR-T therapy in patients were compiled. This included number of enrolled patients, median age, sex ratio, prior treatment rounds, specific CAR-T therapy targets, use of combination therapies, and BTKi application. Genetic and cellular markers such as *TP53* mutations and Ki67 proliferation index were noted, along with central nervous system (CNS) involvement and past transplantation procedures. The primary outcome measures of concern were safety and efficacy.

Two authors (HX Wan and SQ Weng) independently conducted the literature screening based on a predefined search strategy to identify preliminary reports potentially relevant to the topic of study. The screening process strictly adhered to established inclusion and exclusion criteria, ensuring that only studies meeting all criteria were considered for final analysis. The researchers conducted an in-depth full-text review of all initially selected citations and compiled a list of studies that met the eligibility requirements. Any discrepancies in the screening results were resolved through mutual discussion between the researchers, and persistent disagreements were refereed by an external panel of experts.

### Quality assessment

To assess the quality of the literature, the MINORS tool was used, tailored specifically for assessing the methodological quality of nonrandomized studies. The methodological quality of each study was evaluated by categorizing the risk of bias as low or high; scores below 10 indicated a low risk, whereas scores of 10 or greater denoted a high risk.

### Statistical analysis

The cumulative incidence (event rate) and 95% confidence intervals (CIs) were calculated for each specified outcome. The distribution of outcome proportions was validated for normality using the Shapiro–Wilk test, implemented through the “shapiro. test” function in R, aligning with the assumptions of our chosen meta-analytic model. Depending on the I² statistic, a random-effects model was used for I² values > 50% and a fixed-effects model for values ≤ 50%. Subgroup analyses were conducted to investigate the sources of heterogeneity and impact of various factors on treatment efficacy. In addition, we grouped and discussed prospective and retrospective studies to clarify the differences between them. To explore the effects of studies with a high risk of bias, subgroup analyses were performed for studies classified with low or high risks of bias.

This meta-analysis was conducted using the R software (R Foundation for Statistical Computing, Vienna, Austria, version 4.3.3). The “meta” package was used for statistical analysis.

## Results

### Characteristics of included literature

From an initial screening of 1,317 potentially relevant studies, we used EndNote software to preliminarily exclude 174 review articles, 28 case reports, and 131 duplicates. Based on their abstracts and titles, we further excluded 984 studies due to reasons such as basic research, irrelevance to the study topic, duplication, or lack of quantitative data. We conducted a full-text review of 22 studies, excluding those dual-target CAR-T, and case series with fewer than 10 patients. Ultimately, 16 studies (16–30) encompassing 984 patients were included in the analysis (**Supplementary Figure 1**).

Among the included studies, 10 were published in peer-reviewed journals, and 6 were presented at conferences.13 studies employed the Brexu-cel CAR-T cell product (16–25, 28–30), and CTL019 CAR-T cell product (27), Liso-cel CAR-T cell product and mixed CAR-T cell product (26) (including Brexu-cel, Tisa-cel, and Liso-cel) were used in the remain three studies, respectively. The median age were 66.5 years (ranged from 38 to 89) of included patients, and male patients account for 73% of the total population. Most patients were those who had relapsed after multiple lines (ranged from 1 to 12) of therapy, including BTKi treatment. 8 studies also included patients with secondary central nervous system infiltration (19–21, 24, 28–30). Baseline patient characteristics are provided in **Table 1**.

**Table 1:** The clinical characteristics of patients in the studies included in the systematic review.

First author, year	Number of enrolled patients	Age median (range)	Sex male/ female	Prior therapies median (range)	Target antigens	Product	TP53 aberration No. (%)	Ki67≥30% No. (%)	Blastoid/5pleomorphic No. (%)	BTKi history No. (%)	CNS involvement No. (%)	Previous HSCT No. (%)
Wang M, 2023 (16)	68	65 (38-79)	57/11	1 - 5	CD19	Brexu-cel	6 (9%)	40 (59%)	21 (31%)	68 (100%)	NA	43 (29%)
Banerjee T, 2023 (17)	10	72.0 (62–80)	9/1	4 (3-5)	CD19	Brexu-cel	1 (10%)	1 (10%)	5 (50%)	10 (100%)	0 (0%)	2 (20%)
Lacoboni G,2021 (18)	33	67.0 (62-72)	29/4	2 (1-8)	CD19	Brexu-cel	4 (12%)	16 (49%)	8 (24%)	14 (42%)	NA	12 (36%)
Ryan CE, 2023 (19)	10	60.0 (44-79)	5/5	4 (2-6)	CD19	9 Brexu-cel,1Tisa-cel	5 (50%)	5 (50%)	6 (60%)	10 (100%)	10 (100%)	3 (30%)
Wang YC,2022 (20)	168	67.0 (34-89)	128/40	3 (1-10)	CD19	Brexu-cel	81 (48%)	131 (78%)	72 (43%)	144 (86%)	10%	52 (31%)
Locke F,2022 (21)	135	65.6	107/28	4 (2-12)	CD19	Brexu-cel	NA	NA	NA	120 (89%)	5 (4%)	40 (30%)
Hess G, 2023 (22)	111	64.3 (42-80)	91/20	3 (1-9)	CD19	Brexu-cel	24 (22%)	71 (64%)	37 (33%)	111 (100%)	NA	73 (66%)
Herbaux C,2021 (23)	47	67.0 (45-79)	44/3	3 (2-8)	CD19	Brexu-cel	NA	37 (79%)	NA	47 100%	NA	16 (34%)
Rejeski K,2023 (24)	103	66.0 (49-89)	NA	3 (2-4)	CD19	Brexu-cel	43 (42%)	80 (78%)	41 (40%)	NA	5 (4.9%)	33 (32%)
Goy A, 2023 (25)	23	69.0 (43-79)	18/5	4 (1-10)	CD19	Brexu-cel	NA	NA	NA	NA	NA	NA
Wang M 2023 (13)	88	68.5(36-86)	67/21	3(1-11)	CD19	Liso-cel	20 (23%)	66 (75%)	27 (31%)	47 (53%)	7 (8%)	29 (33%)
Ababneh H, 2022 (26)	21	65.0(43-83)	NA	NA	CD19	17 brexu cel, 3 Tisa-cel, and 1 liso-cel	NA	NA	NA	NA	NA	NA
Minson A 2023 (27)	20	66	15/5	2 (1-5)	CD19	CTL019 CAR-T	9 (45%)	NA	3 (15%)	10 (50%)	NA	20 (100%)
Ahmed G, 2024 (28)	12	72 (50-80)	9/3	4 (2-6)	CD19	Brexu-cel	NA	NA	NA	11 (92%)	12 (100%)	NA
O’Reilly MA, 2024 (29)	83	68 (41-78)	60/23	2 (2-7)	CD19	Brexu-cel	32 (38%)	63 (76%)	32 (38%)	83 (100%)	1 (1%)	43 (52%)
Romancik JT,2021 (30)	52	66.0 (47-79)	43/9	3 (2-8)	CD19	Brexu-cel	17 (33%)	43 (83%)	16 (30%)	52 (100%)	7 (13.5%)	23 (44%)

No. (%): Indicates the number of cases or patients with a specific characteristic or event in the table, presented as both an absolute count and a percentage of the total sample size.

BTKi: Represents patients who have previously undergone treatment with Bruton’s tyrosine kinase inhibitors (BTKi).

TP53 aberration No. (%): Indicates patients with abnormalities or mutations in the TP53 gene.

Ki67≥30%: Represents the proportion and count of patients with a Ki67 index greater than or equal to 30%. NA, not available.

In our meta-analysis, we included 16 studies with varied standards for assessing adverse events and efficacy. Specifically, 12 studies utilized the American Society for Transplantation and Cellular Therapy (ASTCT) consensus guidelines for grading CRS and ICANS (17–20, 22–29), 3 studies used the Lee criteria(2014) (16, 21), and 1 study did not clearly specify the adverse event assessment criteria (30). For efficacy assessment, all studies that reported efficacy used the Lugano classification (2014) to determine the response to CAR-T therapy.

### Efficacy

**Figure 1:**
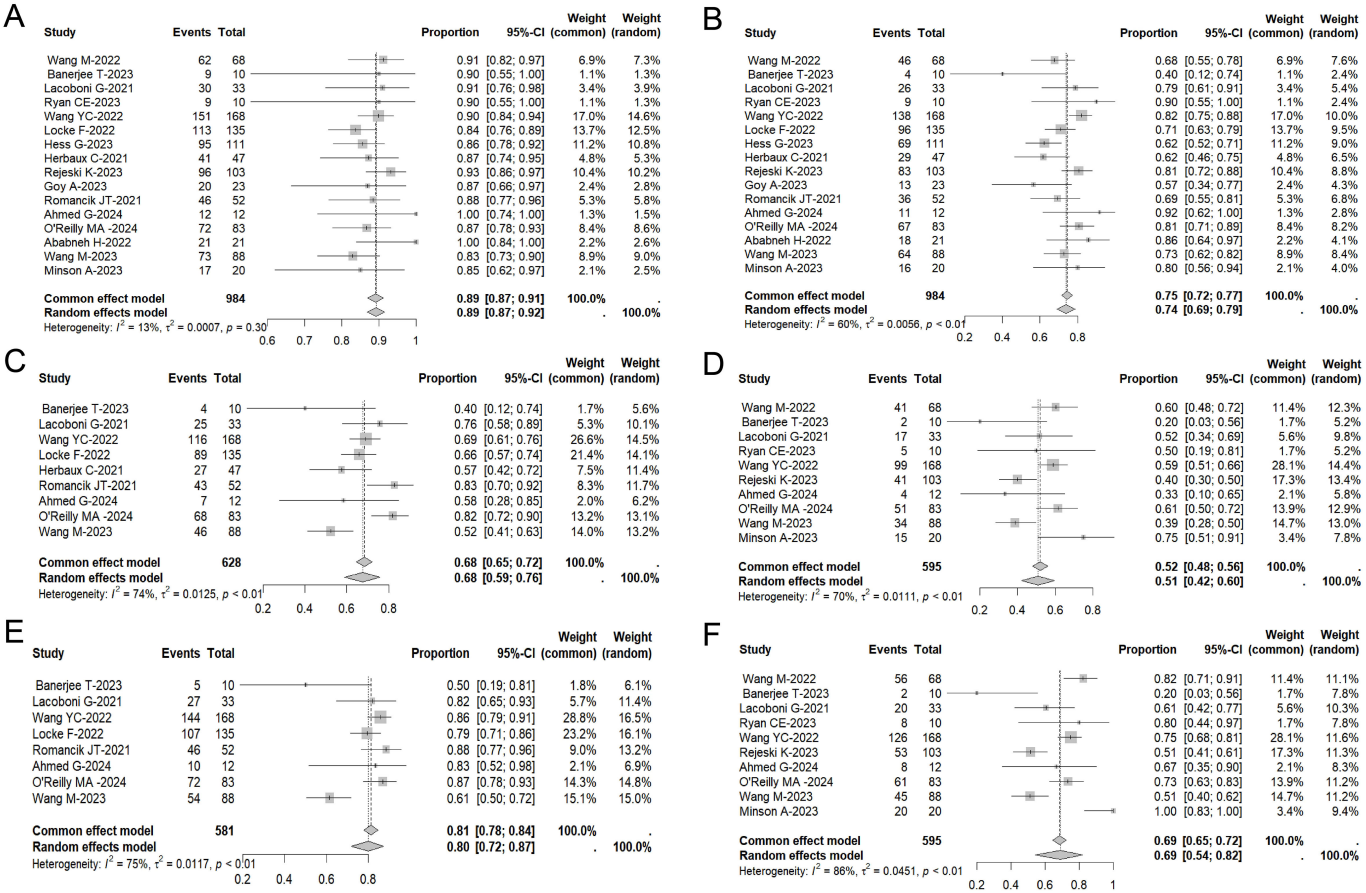
Forest plot of ORR **(A)**, CR rates **(B)**, 6-month-PFS **(C)**, 6-month-OS **(D)**, 1-year PFS rate **(E)** and 1-year OS rate **(F)** in patients with MCL treated with CAR-T therapy across multiple studies.

All studies provided clinical remission data (**Supplementary Table 1**). Among the 984 analyzed patients who received CAR-T therapy for R/R MCL, an ORR of 89% (95% CI: 87%–91%, I²: 13%) was observed (**Figure 1A**). Of these, 74% (95% CI: 69%–79%, I²: 60%) patients attained complete remission (**Figure 1B**). In total, the 6-month PFS rate was 68% (95% CI: 59%–76%, I²: 74%) (**Figure 1C**), the 12-month PFS rate was 51% (95% CI: 42%–60%, I²:70%) (**Figure 1D**). For OS, the 6-month OS rate was 80% (95% CI: 72%–87%, I²: 75%) (**Figure 1E**), whereas the 12-month OS rate remained at 69% (95% CI: 54%–82%, I²: 86%) (**Figure 1F**).

### Safety

**Figure 2:**
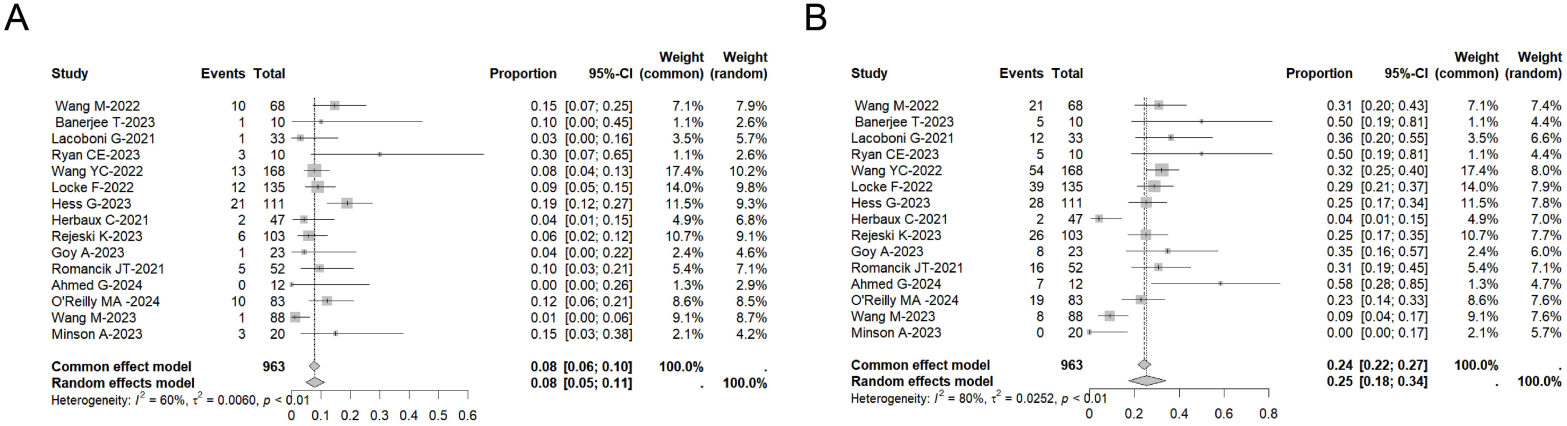
Forest plot of the incidence of ≥ Grade 3 CRS **(A)** and the incidence of ≥ Grade 3 ICANS **(B)** across multiple studies involving CAR-T therapy in MCL patients.

In the pooled analysis of safety data from all treated patients, 86% (95% CI: 81%–91%, I^2:^ 71%) of individuals experienced varying grades of CRS. 8% (95% CI: 5%–11%, I^2^: 60%) of patients had CRS of grade 3 or higher (**Figure 2A**). Additionally, immune effector cell-associated neurotoxicity syndrome (ICANS) was observed in 52% (95% CI: 43%–61%, I^2^: 77%) of 805 assessed patients, with 22% (95% CI: 14%–30%, I^2^: 79%) experiencing ICANS of grade 3 or higher (**Figure 2B**).

### Subgroup analysis

The subgroup analysis of prognostic high-risk factors for MCL indicated varying CR rates among different patient categories. Patients with Ki-67 index below 30% had ORR rate of 87% and CR rate of 65%, whereas those with Ki-67 index of 30% or more had ORR rate of 88% and CR rate of 74%. Patients without and with *TP53* mutations had ORR rate of 95% and 89%, and CR rate of 81% and 70%, respectively. The absence and presence of CNS involvement was associated with ORR rate of 88% and 89%, and CR rate of 72% and 82%, respectively. Nonblastoid/pleomorphic patients and blastoid/pleomorphic patients had ORR rate of 91% and 85%, and CR rate of 75% and 75%, respectively. Patients who had not received prior BTKi treatment exhibited ORR rate of 93% and CR rate of 83%, whereas those with a history of such treatment exhibited ORR rate of 83%, and CR rate of 70%. Patients without a history of hematopoietic stem cell transplantation (HSCT) achieved a CR rate of 70%, while those with a prior HSCT history had a notably higher CR rate of 83%. When stratified by the number of prior lines of therapy, patients who received three or fewer treatments showed a CR rate of 75%, whereas those who had more than three lines of therapy had a slightly lower CR rate of 71% (**Table 2**).

**Table 2:** Subgroup analysis of the impact of biomarkers and clinical features on remission rates in meta-analysis.

Variable	ORR Rate (95%)	I ^2^(%)	P value	CR Rate (95%)	I ^2^(%)	P value
Ki-67 proliferation index						
<30%	0.87 (0.38-0.98)	0	0.98	0.65 (0.44-0.82)	0	0.38
≥30%	0.88 (0.80-0.93)	0		0.74 (0.65-0.82)	0	
TP53 aberration						
No	0.95 (0.70-0.99)	0	0.46	0.81 (0.66-0.90)	76	0.22
Yes	0.89 (0.81-0.94)	0		0.70 (0.61-0.80)	0	
CNS involvement						
No	0.88 (0.76-0.95)	33	0.88	0.72 (0.52-0.85)	81	0.43
Yes	0.89 (0.82-0.92)	0		0.82 (0.59-0.93)	49	
Blastoid/pleomorphic						
No	0.91 (0.86-0.94)	0	0.18	0.75 (0.59-0.86)	56	0.91
Yes	0.85 (0.73-0.92)	50		0.75 (0.59-0.86)	64	
BTKi history						
No	0.93 (0.83-0.97)	0	0.16	0.83 (0.71-0.91)	56	0.05
Yes	0.87 (0.84-0.89)	0		0.70 (0.64-0.76)	64	
Prior HSCT						
No	0.84 (0.73-0.91)	0	0.92	0.70 (0.57-0.80)	0	0.17
Yes	0.83 (0.66-0.93)	0		0.83 (0.66-0.93)	0	
Treatment line						
≤3	0.88 (0.54-0.90)	0	0.39	0.75 (0.69-0.80)	65	0.59
>3	0.86 (0.80-0.90)	0		0.71 (0.54-0.83)	58	

Taking into account the inclusion of different types of CAR-T products, we conducted a subgroup analysis comparing Brexu-cel CAR-T with other CAR-T cell therapies, including Tisa-cel, Liso-cel, and CTL019 CAR-T. Brexu-cel and other CAR-T therapies demonstrated similar ORR and CR rates. Specifically, the ORR was 88% (95% CI: 86%-90%) for Brexu-cel and 87% (95% CI: 62%-96%) for other CAR-T products. The CR rates were 73% (95% CI: 67%-78%) for Brexu-cel and 76% (95% CI: 68%-83%) for others. However, Brexu-cel had a higher incidence of CRS and ICANS, particularly grade 3 ICANS, but also showed better short-term efficacy with higher 6-month PFS and 6-month OS rates (**Supplementary Table 2**).

Due to differences in study design and underlying patient populations, a subset analysis was conducted to compare data from prospective clinical trials with retrospective/case series to identify if differences exist between real-world data and clinical trial data. In the subgroup analysis, retrospective (a total of 456 patients) and prospective (a total of 528 patients) studies were compared. Retrospective studies demonstrated slightly higher ORR and CR rates. Specifically, the ORR was 91% (95% CI: 88%-93%) in retrospective studies and 86% (95% CI: 82%-88%) in prospective studies. The CR rate was 77% (95% CI: 69%-83%) in retrospective studies and 70% (95% CI: 65%-75%) in prospective studies. However, 12-month PFS and 12-month OS were significantly better in prospective studies, with 12-month PFS at 57% (95% CI: 44%-69%) compared to 46% (95% CI: 36%-56%) in retrospective studies, and 12-month OS at 80% (95% CI:54%-94%) compared to 61% (95% CI: 47%-73%) in retrospective studies. The incidence of CRS was similar between the two groups, but Grade 3 CRS was more frequent in prospective studies. ICANS rates were comparable between retrospective and prospective studies (**Supplementary Table 3**).

Among the included studies, 9 were considered to have a low risk of bias and 7 had a moderate risk of bias (**Supplementary Tables 4, 5**). There was no significant correlation between bias risk and outcomes such as disease remission, PFS at 12 months, or adverse events like CRS or ICANS.

## Discussion

The results of this meta-analysis demonstrate that CAR-T cell therapy is an effective treatment for R/R MCL with manageable safety. In the 16 studies included, a total of 984 patients received CAR-T therapy, achieving an ORR of 89% and a CR rate of 74%. Additionally, the 6-month and 12-month PFS rates were 69% and 53%, respectively, while the OS rates were 80% and 69%, respectively. Although 8% of patients experienced grade 3 or higher CRS and 22% experienced grade 3 or higher ICANS, overall, CAR-T therapy demonstrated promising efficacy and manageable adverse effects in patients with R/R MCL.

Over the past 10 years, BTKis have greatly improved the treatment outcome of patients with R/R MCL. However, a considerable number of patients still experience relapse and poor outcomes after BTKi therapy. Therefore, it is important to explore alternative treatment options for patients with MCL that are resistant to BTKis. A study compared the OS of patients with R/R MCL with failed covalent BTKi treatment. The patients were either treated with standard of care or Brexu-cel (31, 32). The results indicated that patients who received Brexu-cel had a significantly higher OS rate compared with those who received standard of care treatment.

We conducted a detailed search of the database, which currently encompassing numerous clinical trials of CAR-T cell products for MCL. Due to the limited number of studies on bispecific and other targets of CAR-T therapy for MCL, and differences in targets and manufacturing processes compared to CD19 single-target CAR-T, this study excluded dual-target and case series with fewer than 10 patients to reduce heterogeneity and bias. Therefore, our article mainly represents the efficacy of CD19 CAR-T therapy for R/R MCL, primarily Brexu-cel. This may not be representative of other CAR-T products. In a subgroup analysis of different CD19 CAR-T therapies, the ORR for the Brexu-cel product was 88% with a CR rate of 73%, while other CAR-T products had an ORR of 87% and a CR rate of 76%. Brexu-cel product and other CAR-T products exhibited similar effectiveness. However, the literature of other CAR-T is limited, these results should be taken with caution.

In addition, this study thoroughly examined various factors that impact CAR-T therapy for MCL. The findings suggested that CAR-T therapy can be effective even in high-risk patients with MCL with characteristics such as TP53 lesions (17p13 deletions or TP53 mutations), blastoid/pleomorphic histology, CNS involvement, high Ki-67 index, and complex karyotype. These indicating CAR-T have a wider applicability in MCL and suggesting CAR-T maybe a promising therapeutic approach for MCL patients. Notably, results showed prior exposure to BTKis may be linked to lower CR rates. In some way, it indicated early adoption of CAR-T therapy may result in better treatment outcomes.

Patients with MCL that has spread to the CNS typically have a poor prognosis and limited treatment options, with a median OS of less than 5 months (33, 34). Recent studies have reported that CAR T-cell therapy may be a promising treatment option for MCL with CNS involvement (35). In our meta-analysis, 8 studies encompassed patients with CNS involvement. The aggregated results from our meta-analysis demonstrated a remarkable ORR of 89% and a CR rate of 82% for R/RMCL with CNS involvement, indicating significant efficacy of CAR-T cells in treating R/R MCL. However, further prospective clinical trials are needed to confirm these encouraging results.

There are certain differences among various adverse event grading standards (36). Among the 16 articles in our study, 13 used the ASTCT criteria. We compared the different grades of CRS and ICANS between the two standards and found minimal differences in grade 3 or higher adverse events (36, 37). Therefore, we discussed the incidence of adverse events across all articles together. Additionally, a stratified analysis of articles using different assessment criteria showed consistent evaluation results (data not shown). In conclusion, different grading standards have little impact on the safety assessment in this study.

There were some limitations in this study. Firstly, the CAR-T cells used in included articles were mainly Brexu-cel, indicating our article mainly represents the efficacy of Brexu-cel for R/R MCL, and may not be representative of other CAR-T products. Secondly, due to the median PFS and OS not being reached in some clinical studies, coupled with the likely missing follow-up data from many real-world, non-clinical trial studies, there may be biases in the analysis of follow-up data. This may lead to systematic biases in the follow-up data, affecting the accuracy and reliability of the results. Nonetheless, to the best of our knowledge, this is the first comprehensive analysis of the efficacy and safety of CAR-T therapy in treating MCL, as well as exploration of high-risk factors. This study with largest sample size would provide more reliable information for CAR-T cell in MCL.

In conclusion, CAR-T therapy is highly effective as a salvage treatment for R/R MCL, even in patients with high-risk features. However, due to the lack of longer follow-up, the long-term efficacy remains to be determined. The variability in patient responses underscores the importance of personalized treatment approaches. Future research should focus on long-term efficacy assessments to optimize treatment strategies and improve overall patient outcomes.

## Data Availability

The original contributions presented in the study are included in the article/**Supplementary Material**. Further inquiries can be directed to the corresponding authors.

## Supplementary material

Supplementary Figure 1 The flow chart of literature screening.
