# Supplementary figures and images for: Chimeric antigen receptor T-cell therapy in relapsed or refractory mantle cell lymphoma: a systematic review and meta-analysis
Source: Front Immunol. 2024 Sep 6;15:1435127. doi: 10.3389/fimmu.2024.1435127 (PMC11412868; doi:10.3389/fimmu.2024.1435127)

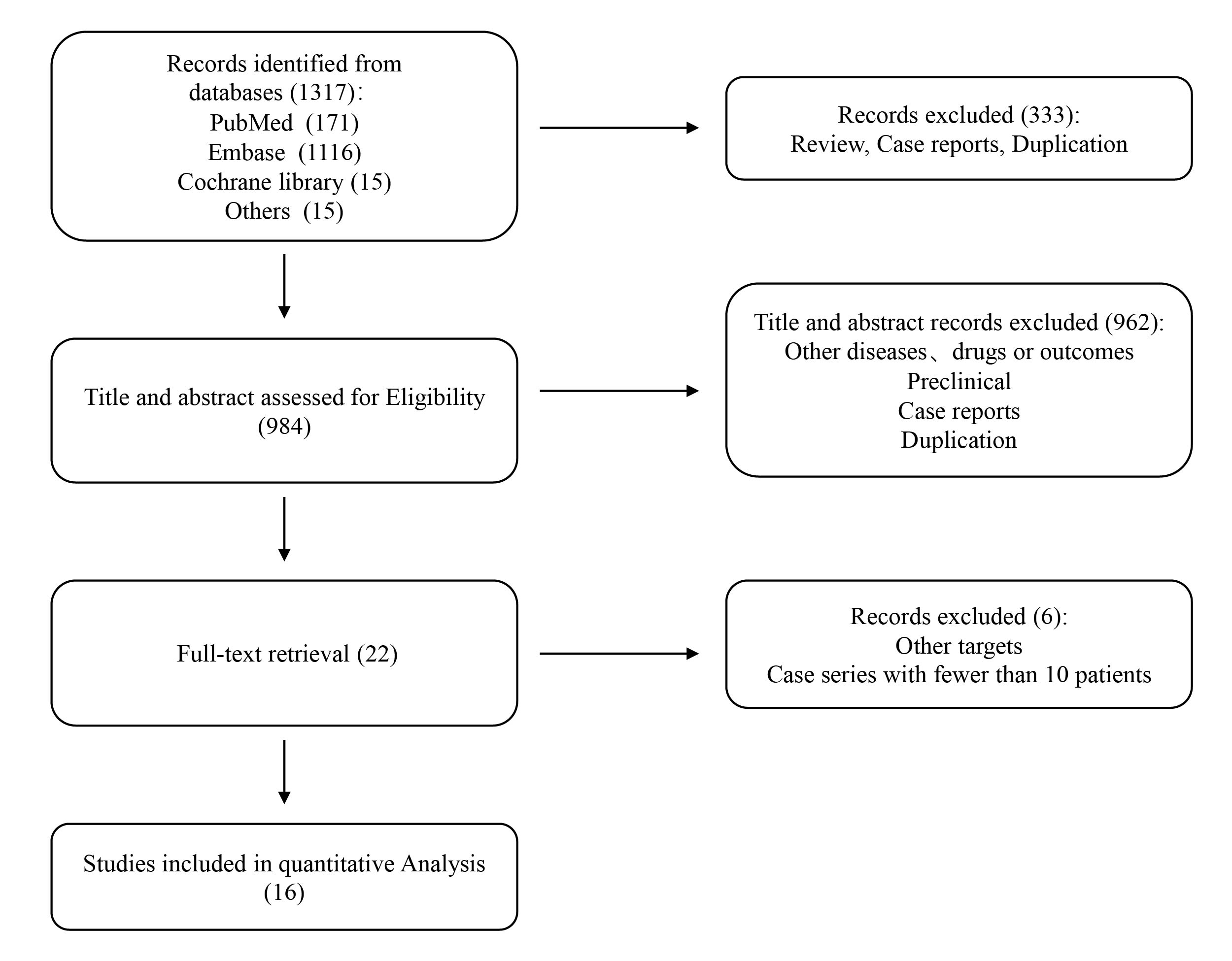

Supplement: Supplementary Figure 1 — The flow chart of literature screening. [file Image1.jpeg]
