# Supplementary material for: Chimeric antigen receptor T-cell therapy in relapsed or refractory mantle cell lymphoma: a systematic review and meta-analysis
Source: Front Immunol. 2024 Sep 6;15:1435127. doi: 10.3389/fimmu.2024.1435127 (PMC11412868; doi:10.3389/fimmu.2024.1435127)
Supplement: Supplementary file 2 [file Table1.docx]

Table S1: Efficacy and safety evaluation data

| First Author,  year | Number  of  enrolled  patients | ORR | CR | CRS | ICANS | ≥3 Grade CRS | ≥3 Grade ICANS | PFS 6m | PFS 12m | OS 6m | OS 12m |
| --- | --- | --- | --- | --- | --- | --- | --- | --- | --- | --- | --- |
| Wang M,  2022 | 68 | 62(91%） | 46(68%) | 62(91%) | 43(63%) | 10(7%) | 21(31%) | NA | 41(60%) | NA | 56(82%) |
| Banerjee T,  2023 | 10 | 9(90%) | 4(40%) | 9(90%) | 7(70%) | 1(10%) | 5(50%) | 4(40%) | 2(20%) | 5(50%) | 2  (20%) |
| Lacoboni G,  2021 | 33 | 30(91%） | 26(79%） | 30(91%) | 21(64%) | 1(3%) | 12(36%) | 25(76%) | 17(52%) | 27(82%) | 20(61%) |
| Ryan CE, 2023 | 10 | 9(90%) | 9(90%) | 9(90%) | 7(70%) | 3(30%) | 5(50%) | NA | 5(50%) | NA | 8(80%) |
| Wang YC,  2022 | 168 | 151(90%） | 138(82%） | 151(90%） | 102(61%) | 13(8%) | 54(32%) | 116(69%) | 99(59%) | 144(86%) | 126(75%) |
| Locke F,2022 | 135 | 113(84%) | 96(71%) | NA | NA | 12(9%) | 39(29%) | 89(66%) | NA | 107(79%) | NA |
| Hess G, 2023 | 111 | 95(86%) | 69(62%) | 95(86%) | 57(51%) | 21(19%) | 28(25%) | NA | NA | NA | NA |
| Herbaux C,2021 | 47 | 41(87%） | 29(62%) | 37(79%) | 23(49%) | 2(4%) | 2(4%) | 27(57%) | NA | NA | NA |
| Rejeski K,2023 | 103 | 96(93%) | 83(81%) | 92(89%) | 64(62%) | 6(6%) | 26(25%) | NA | 41(40%) | NA | 53（51%) |
| Goy A, 2023 | 23 | 20(87%） | 13(57%） | NA | NA | 1(4%) | 8(35%) | NA | NA | NA | NA |
| Wang M  2023 | 88 | 73(83%) | 64(73%) | 54(61%) | 27(31%) | 1(1%) | 8(9%) | 46(52%) | 34（39%） | 54（61%） | 45（51%） |
| Ababneh H, 2022 | 21 | 21(100%) | 18(86%) | NA | NA | NA | NA | NA | NA | NA | NA |
| Minson A  2023 | 20 | 17（85%） | 16（80%） | 15（75%） | 2（10%） | 3（15%） | 0 | NA | 15（75%） | NA | 20(100%) |
| Ahmed G  ，2024 | 12 | 12(100%) | 11(92%) | 11(92%) | 10(83%) | 0(0%) | 7(58%) | 7(58%) | 4(33%) | 10(75%) | 8(63%) |
| O'Reilly MA  ，2024 | 83 | 72(87%) | 67(81%) | 77(93%) | 46(55%) | 10(12%) | 19(22%) | 68(82%) | 51(62%) | 72(87%) | 61(74%) |
| Romancik JT,2021 | 52 | 46(88%） | 36(69%) | 44(85%) | 30(58%) | 5(10%) | 16(31%) | 43(83%) | NA | 46(88%) | NA |

PFS 6m: 6-month progression-free survival. PFS 12m: 12-month progression-free survival. OS 6m: 6-month overall survival. OS 12m: 12-month overall survival.

图六

图五

111

Table S2: Subgroup analysis differentiates the outcome from Brexu-cel and other CAR-T products

| Outcome | Brexu-cel (N=855) | Others (N=129) | P-value |
| --- | --- | --- | --- |
| ORR | 0.88(0.86-0.90) | 0.87 (0.62-0.96) | 0.86 |
| CR | 0.73 (0.67-0.78) | 0.76 (0.68-0.83) | 0.5 |
| CRS | 0.89 (0.89; 0.91) | 0.64 (0.54; 0.74) | 0.001 |
| ICANS | 0.59 (0.55; 0.62) | 0.24 (0.10; 0.46) | 0.004 |
| CRS3 | 0.09 (0.07; 0.10) | 0.10 (0.06; 0.16) | 0.38 |
| ICANS3 | 0.29 (0.24; 0.35) | 0.07 (0.04; 0.14) | 0.0001 |
| PFS6 | 0.70(0.62; 0.77) | 0.52 (0.42; 0.62) | 0.006 |
| PFS12 | 0.51 (0.43; 0.59) | 0.55 (0.29; 0.79) | 0.79 |
| OS6 | 0.83 (0.80; 0.87) | 0.61 (0.51; 0.71) | 0.001 |
| OS12 | 0.67 (0.55; 0.77) | 0.94 (0.07; 0.10) | 0.46 |

Table S3: Subgroup analysis differentiates the outcome from retrospective and prospective studies

| Outcome | Retrospective (N=456) | Prospective (N=528) | P-value |
| --- | --- | --- | --- |
| ORR | 0.91(0.88; 0.93) | 0.86(0.82; 0.88) | 0.01 |
| CR | 0.77(0.69; 0.83) | 0.70(0.65; 0.75) | 0.16 |
| CRS | 0.88(0.85; 0.91) | 0.84(0.72; 0.92) | 0.39 |
| ICANS | 0.61(0.56; 0.65) | 0.42(0.50; 0.60) | 0.05 |
| CRS3 | 0.07(0.05; 0.10) | 0.10(0.06; 0.16) | 0.36 |
| ICANS3 | 0.31(0.20; 0.45) | 0.21(0.14; 0.31) | 0.18 |
| PFS 6m | 0.68[0.58; 0.77) | 0.68(0.52; 0.80) | 0.97 |
| PFS 12m | 0.46(0.36; 0.57) | 0.57(0.44; 0.69) | 0.2 |
| OS 6m | 0.84(0.80; 0.88) | 0.77(0.63; 0.87) | 0.2 |
| OS 12m | 0.61(0.47; 0.73) | 0.80(0.54; 0.94) | 0.17 |

PFS 6m: 6-month progression-free survival. PFS 12m: 12-month progression-free survival. OS 6m: 6-month overall survival. OS 12m: 12-month overall survival.

Table S4: Meta-Analysis study quality assessment based on MINORS criteria

| Author;Year | The purpose of the research is clearly given | Continuity of inclusion of patients | Collection of anticipated date | The end points reflect the study objective appropriately | The objectivity of the evaluation of end points | Whether the follow-up time is sufficient | The loss of follow-up rate is less than 5% | Whether the sample size was estimated |
| --- | --- | --- | --- | --- | --- | --- | --- | --- |
| Wang M, 2022 | 2 | 2 | 2 | 2 | 1 | 2 | 2 | 0 |
| Banerjee T, 2023 | 2 | 1 | 2 | 2 | 1 | 2 | 2 | 0 |
| Lacoboni G,2021 | 2 | 2 | 2 | 2 | 1 | 2 | 1 | 0 |
| Goy A, 2023 | 2 | 1 | 1 | 1 | 1 | 2 | 1 | 0 |
| Ryan CE, 2023 | 2 | 2 | 2 | 1 | 1 | 1 | 2 | 0 |
| Wang YC,2022 | 2 | 2 | 2 | 2 | 1 | 2 | 1 | 0 |
| Romancik JT,2021 | 2 | 1 | 1 | 1 | 0 | 0 | 1 | 0 |
| Locke F,2022 | 2 | 1 | 1 | 1 | 1 | 1 | 1 | 0 |
| Hess G, 2023 | 2 | 2 | 1 | 1 | 1 | 1 | 1 | 0 |
| Herbaux C,2021 | 2 | 1 | 1 | 1 | 1 | 1 | 1 | 0 |
| Rejeski K,2023 | 2 | 1 | 1 | 1 | 1 | 1 | 1 | 0 |
| Ahmed G,2024 | 2 | 1 | 2 | 2 | 1 | 2 | 2 | 0 |
| O'Reilly MA,2024 | 2 | 2 | 2 | 2 | 1 | 2 | 2 | 0 |
| Wang M,2023 | 2 | 2 | 2 | 2 | 2 | 1 | 2 | 0 |
| Wang M,2023 | 2 | 2 | 2 | 2 | 2 | 1 | 2 | 0 |
| Minson A, 2023 | 2 | 2 | 2 | 2 | 1 | 1 | 2 | 0 |

Table S5: Risk of bias assessment in studies using MINORS criteria

| Author | Year | Study design | Sample size | Biased risk assessment |
| --- | --- | --- | --- | --- |
| Wang M | 2022 | Cross-sectional study | 68 | Low |
| Banerjee T | 2023 | Case series | 10 | Low |
| Lacoboni G | 2021 | Cross-sectional study | 33 | Low |
| Goy A | 2023 | Cross-sectional study | 23 | Median |
| Ryan CE | 2023 | Case series | 10 | Low |
| Wang YC | 2022 | Cross-sectional study | 168 | Low |
| Romancik JT | 2021 | Cross-sectional study | 52 | Median |
| Locke F | 2022 | Cross-sectional study | 135 | Median |
| Hess G | 2023 | Cross-sectional study | 111 | Median |
| Herbaux C | 2021 | Cross-sectional study | 47 | Median |
| Rejeski K | 2023 | Cross-sectional study | 103 | Median |
| Ahmed G | 2024 | Case series | 12 | LOW |
| O'Reilly MA | 2024 | Cross-sectional study | 83 | LOW |
| Ababneh H | 2022 | Cross-sectional study | 21 | Median |
| Wang M | 2023 | Cross-sectional study | 88 | Low |
| Minson A | 2023 | Case series | 20 | Low |
