# Supplementary material for: Chimeric antigen receptor T-cell therapy in relapsed or refractory mantle cell lymphoma: a systematic review and meta-analysis
Source: Front Immunol. 2024 Sep 6;15:1435127. doi: 10.3389/fimmu.2024.1435127 (PMC11412868; doi:10.3389/fimmu.2024.1435127)
Supplement: Supplementary file 3 [file DataSheet1.docx]

Search strategy and selection criteria

((((Lymphoma, Mantle-Cell[MeSH Terms]) OR (Mantle Cell Lymphoma[Title/Abstract])) OR (Mantle Zone Lymphoma[Title/Abstract])) OR (Lymphoma, Mantle Cell[Title/Abstract])) AND ((((immunotherapy, adoptive[MeSH Terms]) OR (CAR T-Cell Therapy[Title/Abstract])) OR (Therapy, CAR T-Cell[Title/Abstract])) OR (Chimeric Antigen Receptor Therapy[Title/Abstract]))
